# Three-dimensional friction measurement during hip simulation

**DOI:** 10.1371/journal.pone.0184043

**Published:** 2017-09-08

**Authors:** Robert Sonntag, Steffen Braun, Loay Al-Salehi, Joern Reinders, Ulrike Mueller, J. Philippe Kretzer

**Affiliations:** Laboratory of Biomechanics and Implant Research, Clinic for Orthopedics and Trauma Surgery, Heidelberg University Hospital, Heidelberg, Germany; University of Memphis, UNITED STATES

## Abstract

**Objectives:**

Wear of total hip replacements has been the focus of many studies. However, frictional effects, such as high loading on intramodular connections or the interface to the bone, as well as friction associated squeaking have recently increased interest about the amount of friction that is generated during daily activities. The aim of this study was thus to establish and validate a three-dimensional friction setup under standardized conditions.

**Materials and methods:**

A standard hip simulator was modified to allow for high precision measurements of small frictional effects in the hip during three-dimensional hip articulation. The setup was verified by an ideal hydrostatic bearing and validated with a static-load physical pendulum and an extension-flexion rotation with a dynamic load profile. Additionally, a pendulum model was proposed for screening measurement of frictional effects based on the damping behavior of the angular oscillation without the need for any force/moment transducer. Finally, three-dimensional friction measurements have been realized for ceramic-on-polyethylene bearings of three different sizes (28, 36 and 40 mm).

**Results:**

A precision of less than 0.2 Nm during three-dimensional friction measurements was reported, while increased frictional torque (resultant as well as taper torque) was measured for larger head diameters. These effects have been confirmed by simple pendulum tests and the theoretical model. A comparison with current literature about friction measurements is presented.

**Conclusions:**

This investigation of friction is able to provide more information about a field that has been dominated by the reduction of wear. It should be considered in future pre-clinical testing protocols given by international organizations of standardization.

## Introduction

To date, wear of total joint replacements has been the focus of many clinical evaluations and pre-clinical testing which is commonly conducted by joint simulators that can replicate standardized walking cycles. Nevertheless, Sir John Charnely’s approach of reducing friction by using a small head and ultra-high molecular weight polyethylene (UHMWPE) as a bearing material successfully resulted in low frictional forces and torques [1]. With the osteolytic potential of UHMWPE wear particles, the evaluation of new bearing materials and implant designs was chiefly addressed by wear measurements from mechanical in vitro simulation. Using these methods, the conventional UHMWPE has been improved as our modern form of cross-linked and stabilized polyethylene (XPE) within the past few decades [2].

However, head sizes have continuously increased because of the potential for a decreased risk in dislocation [3, 4] and an increase in range of motion [5]. Due to the use of larger head sizes, friction has become more relevant for implant interfaces between modular components [6] and the implant fixation to bone [7]. Nevertheless, the design and dimensions of the intermodular taper interfaces have not been adapted to fit the larger head diameters and to incorporate modern material combinations. This can cause micromotions in the taper connection, which may lead to fretting. Consequently, there is an increasing number of reports on taper wear for implant systems that use large head hard-on-hard bearings, which are associated with tribo-corrosion in the connective interface [6, 8, 9].

It has been shown that not all potential stress factors described clinically are covered in standard testing according to ISO 14242–1. For example, the varying activities in daily life [10] as well as severe conditions that highly stress the bearing components, e.g. implant malpositioning [11, 12], are not simulated but may have a direct influence on the creation of frictional torque. It has been observed that contact pressure and the formation of a load-bearing fluid film play an important role in the technical performance of hard-on-hard bearings, not only for the amount of wear, but also for the level of frictional loading which is generated during articulation [13]. Thus, a freely programmable simulator would allow for the testing of scenarios that are clinically relevant and may provoke high frictional torques.

Historically, mechanical pendulums were used to quantify frictional effects in the natural human joint [14, 15]. These experiments were also implemented by Charnley later on during evaluation of polytetrafluoroethylene (PTFE) and UHMWPE as potential bearing materials in the 1960s [16, 17]. To date, uniaxial experimental setups still represent a standard for friction measurement of ball joints, either under a free or driven rotation and constant or varying axial loads [18–24]. However, this is a simplified approach that reduces the complex gait cycle to only one single rotation (typically extension-flexion). These measurements may be applicable to isolated questions but are limited in regard to the three-dimensional joint motion that is present during daily activities [25].

Online friction measurements have been performed in wear simulators (orbital bearing type according to ISO 14242–3 and standard wear-testing machines according to ISO 14242–1) using a multi-axis machine transducer [26] or custom-made measurement devices [27–31]. In these cases, three-dimensional friction results are mainly validated through a comparison with the existing literature.

Up until this point, friction has been considered from a more academic point of view than for safety reasons during implant development. However, due to recent problems in metal-on-metal bearings, friction is becoming increasingly relevant [32] for more than just all-metal bearings. Measurements are somewhat difficult to take, and thus, friction setups for either one or more complex rotations differ widely, as do their results. Additionally, direct validation of the friction setups is mostly lacking. The purpose of this study was to develop a friction measurement setup in a hip simulator that is typically used for wear evaluation. Verification and validation of the measurements has been performed by:

1. *Repeated* measurements of one single sample with respective test chamber dismantling and re-setup.
2. Use of a *hydrostatic bearing* representing the ‘perfectly lubricated’ hip joint in order to account for any systemic effects.
3. Comparison of friction measurements in the simulator using only the extension-flexion channel with those from a *physical pendulum* under a static load: investigation of measured frictional torque and calculation based on angular damping.
4. *Extension-flexion oscillation under a dynamic force profile* similar to frictional measurements recently published [33].

In addition, more complex friction measurements have been realized and compared to published data from literature.

5. *Three-dimensional friction measurements in the hip simulator* under the standard ISO profile of ‘Normal walking’ for different head diameters of ceramic-on-XPE hip bearings.

## Materials and methods

**Fig 1:**
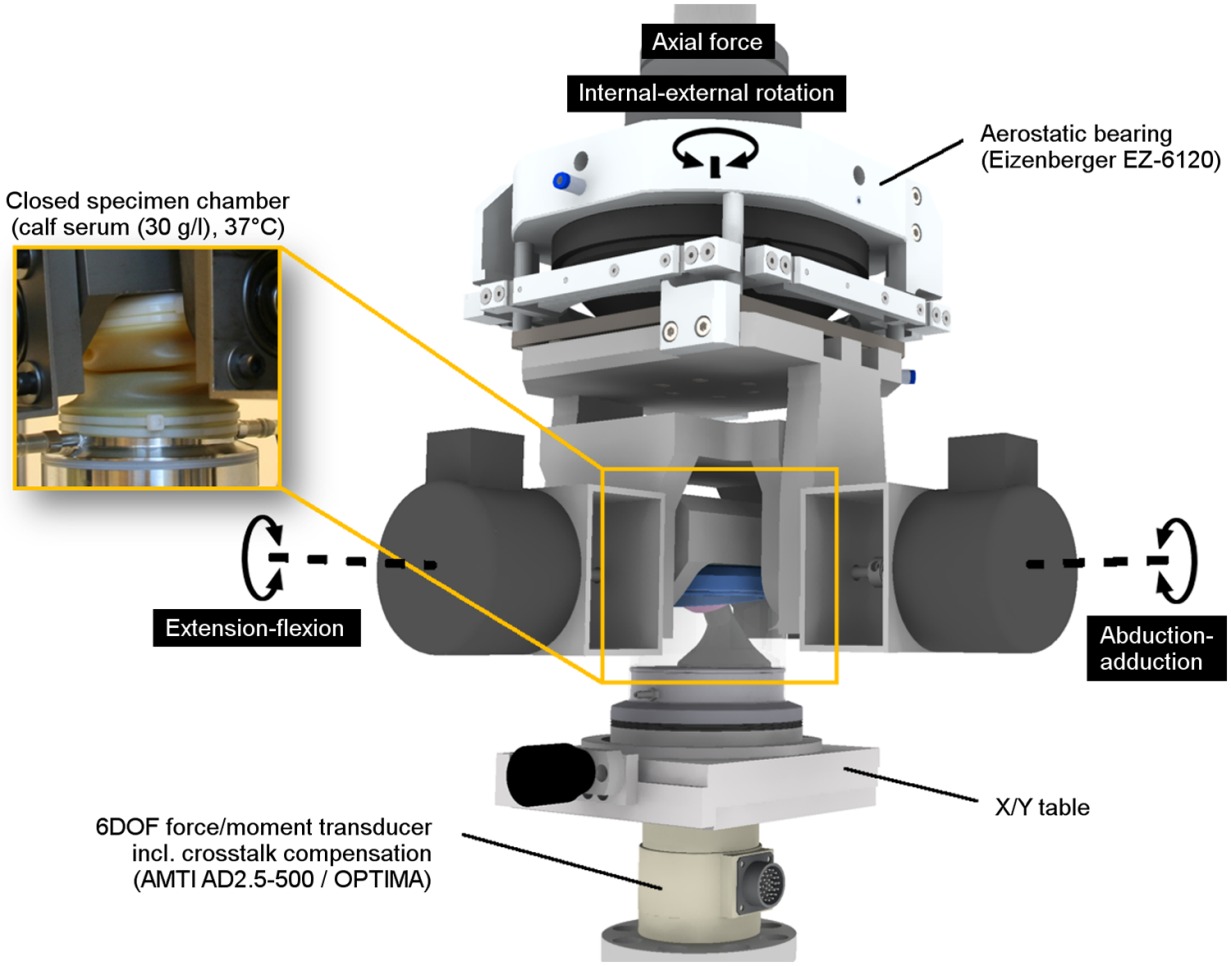
Heidelberg friction simulator. Modification of a single-station simulator to allow for low friction measurements in the hip joint.

A standard one-station hip simulator (Minibionix 852 incl. 4 DOF Hip, MTS Systems Corporation, USA) was modified to allow for friction measurement (Fig 1). In this setup, the head remained still while all rotations (extension-flexion, abduction-adduction, internal-external rotation) were conducted by the acetabular component.

**Fig 2:**
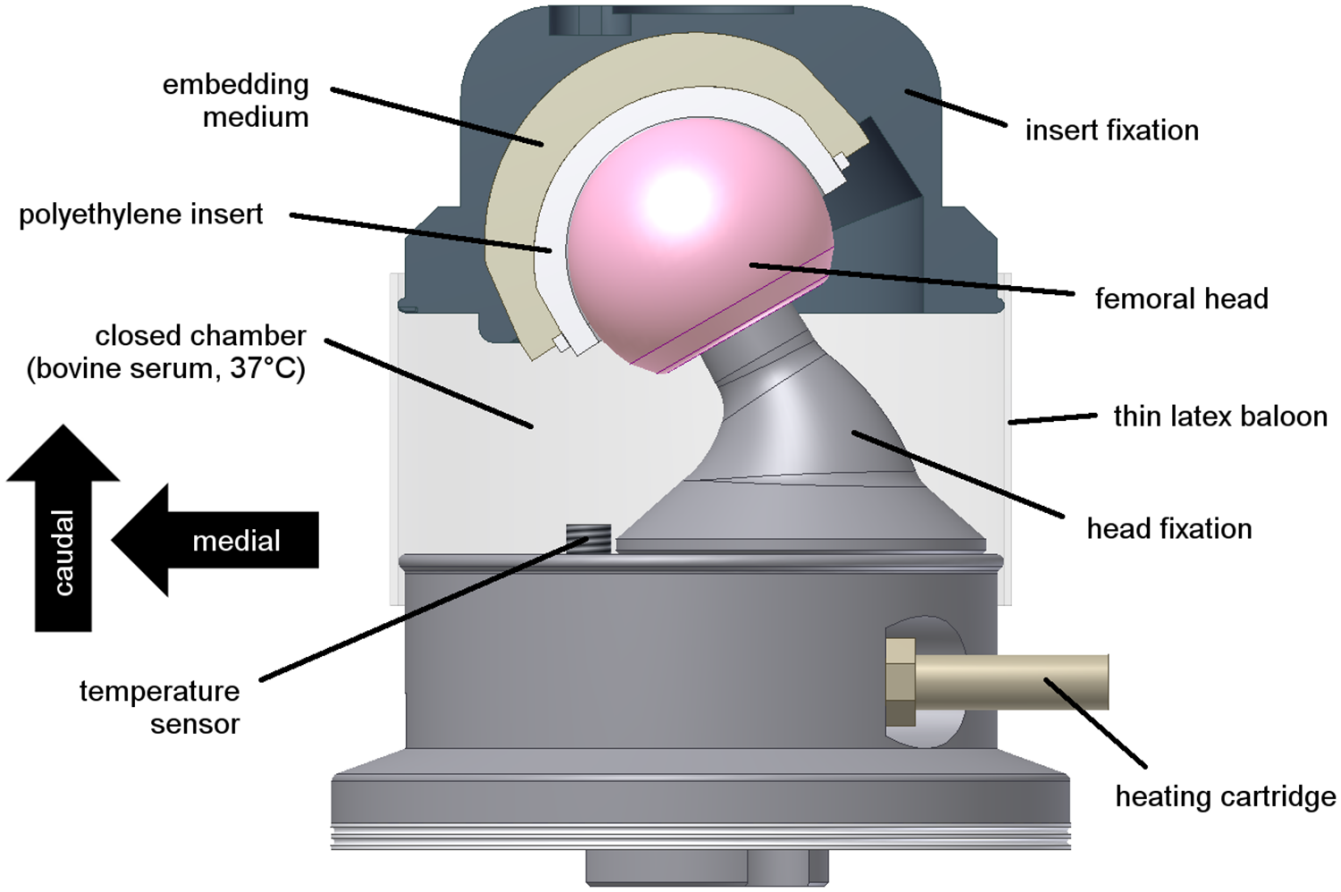
Closed test chamber. Filled with diluted calf serum (protein content: 30 g/l) and temperature-controlled at 37±1°C (36 mm ceramic-on-XPE bearing, right hip).

The bearing was mounted in a closed chamber (loose natural latex balloon of 0.2–0.4 mm thickness) containing constantly temperature-controlled calf serum (37°C ± 0,2°C, 100–110 ml) with a protein content of 30 g/l (according to ISO 14242–1) enriched with 5.85 g/l ethylenediaminetetraacetic acid (EDTA) disodium salt dihydrate and 1.85 g/l sodium azide. The length of the balloon was chosen so that it would be loose during the whole motion cycle in order not to generate any confounding effects. Tests were run under an ideal clinical cup inclination of 45 degrees, corresponding to a technical inclination of 33 degrees relative to the force axis (Fig 2). The femoral head was mounted at an angle of 30 degrees to the vertical axis.

Small frictional forces and torques were intended to be measured during simulation, and thus, it was necessary to reduce systemic disturbances to a minimum. As specimen orientation always requires tolerances, lateral force compensation was mandatory. Roller bearings, which are often used, are already inducing friction in the expected measurement range. Thus, a quasi-frictionless aerostatic bearing (EZ 6120, Eizenberger Luftlagertechnik GmbH, Germany) was utilized in order to eliminate any constraining forces generated due to misalignment of the actuators’ centers of rotation, in respect to the implant’s center of rotation (Fig 1). A custom-made cage allowed for free anterior-posterior and medio-lateral translation of the acetabular component during separation of the aerostatic bearing partners by using a load-bearing air film while all torques were blocked.

The measurement device that detected frictional effects of a well-functioning artificial hip also had to be considered. These effects were expected to be about three orders of magnitude smaller than the resultant joint load and therefore, crosstalk compensation was mandatory. This had been done previously by decoupling the joint load from the frictional torque measurement [31, 33]. In this study, forces and torques were measured in all six degrees of freedom (DOF) by a high-precision multi-axes transducer (AD2.5–500, AMTI, USA). The transducer was calibrated for multiple loads over the whole working range, at 49 points in quarter inch increments on its top surface, using a precision machine (absolute position accuracy of 0.005 mm). This precision calibration allowed for digital signal conditioning and crosstalk compensation at 1024 Hz by a fully programmable amplifier (OPTIMA Signal Conditioner, AMTI, USA). The capture system was calibrated for force and torque output accuracy more precisely than ±0.02% of full scale (F_x_ = F_y_ = 1112 N, M_x_ = M_y_ = 56 Nm, M_z_ = 28 Nm) and ±0.1% of applied load including crosstalk effects.

Heads and cup inserts were mounted inside the fixation devices by 2–3 firm hammer blows. Additionally, the system was loaded up to 3.5 kN three times to allow for any implant seating prior to testing. The center of rotation of the hip bearing was set in the vertical axis of the transducer by a translational X/Y table eliminating confounding torques initiated by high axial forces during hip simulation [34]. This was done by using iterative loading and translation of the center of the head until the measured torques, M_x_ andM_y_, were eliminated.

All measurements were taken under temperature-controlled conditions to address the thermal sensitivity of the measurement devices. Before testing, the articulation partners were separated and all measurement channels were auto-zeroed.

**Fig 3:**
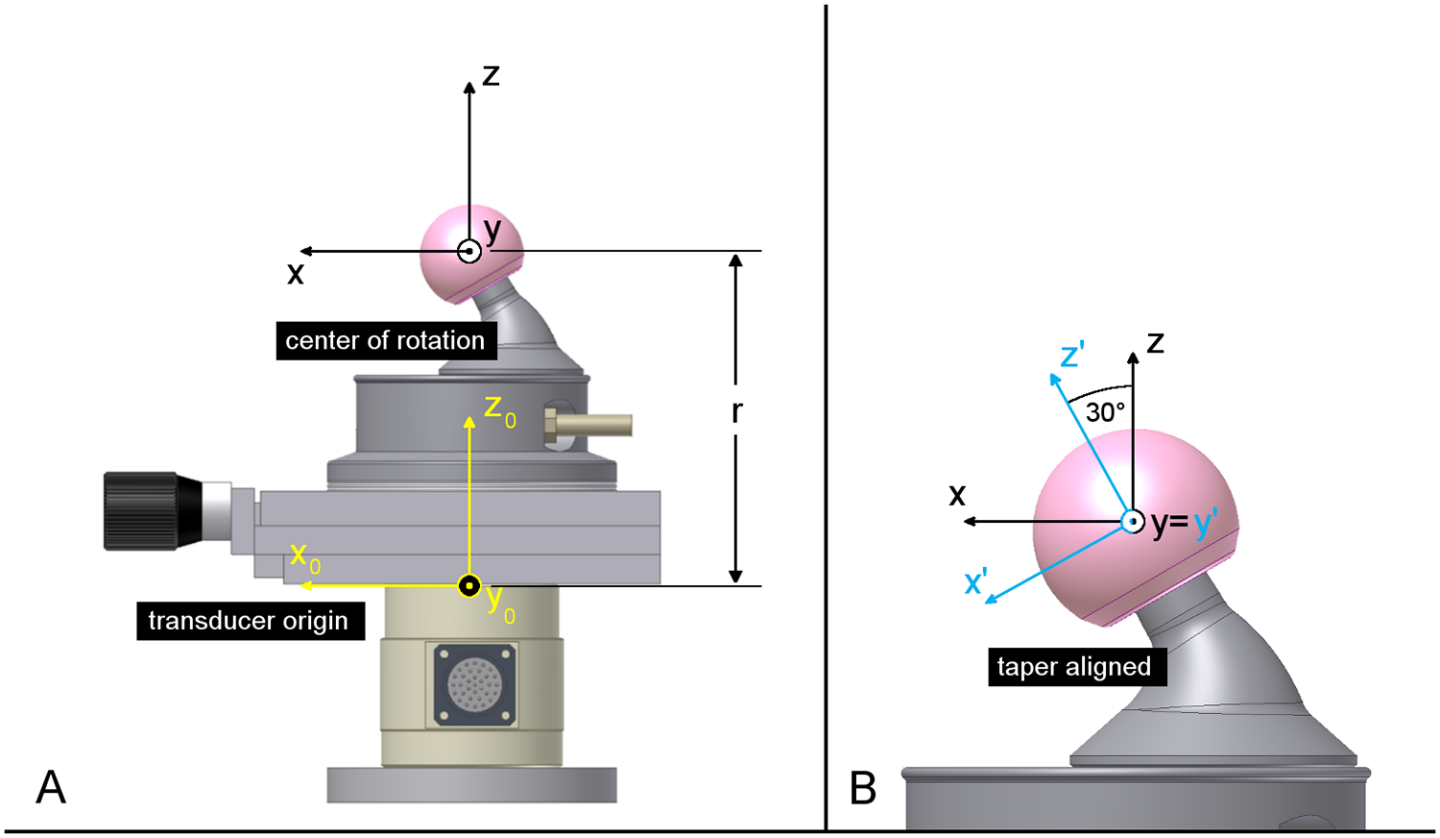
Coordinate transformations. (A) Translation from the transducer origin to the head’s center. (B) Rotation around the y-axes for alignment with the taper axes.

Post-processing of the force and torque data was carried out by a custom-made Matlab routine (7.10.0.499 R2010a, The MathWorks, USA). After converting voltage data to SI units and decreasing the data rate to 256 Hz, forces and torques were transformed from the origin of the transducer (x_0_-y_0_-z_0_ coordinate system) to the head’s center (distancer, x-y-z coordinates) (Fig 3A). The displacement matrix ${\bar{M}}_{displ}$ for torque transformation is given as follows:

$${\bar{M}}_{displ}=r\times F=\left(\begin{array}{c} \Delta x\\ \Delta y\\ \Delta z \end{array}\right)\times \left(\begin{array}{c} F_{x}\\ F_{y}\\ F_{z} \end{array}\right)=\left(\begin{array}{c} -\Delta zF_{y}\\ \Delta zF_{x}\\ 0 \end{array}\right)$$ (1)

where Δx = Δy = 0 (exact positioning by the X/Y table prior to simulation). Subsequently, the torques’ vector $\bar{M}$ and the force vector $\bar{F}$ in the head’s center are calculated as

$$\bar{M}=\left(\begin{array}{c} M_{x}\\ M_{y}\\ M_{z} \end{array}\right)={\bar{M}}_{0}-{\bar{M}}_{displ}=\left(\begin{array}{c} M_{x_{0}}+\Delta zF_{y}\\ M_{y_{0}}-\Delta zF_{x}\\ M_{z_{0}} \end{array}\right)\;with\;\bar{F}=\left(\begin{array}{c} F_{x}\\ F_{y}\\ F_{z} \end{array}\right)=\left(\begin{array}{c} F_{x_{0}}\\ F_{y_{0}}\\ F_{z_{0}} \end{array}\right)$$ (2)

where ${\bar{M}}_{0}=\left(\begin{array}{c} M_{x0}\\ M_{y0}\\ M_{z0} \end{array}\right)$ is the torque vector and ${\bar{F}}_{0}=\left(\begin{array}{c} F_{x0}\\ F_{y0}\\ F_{z0} \end{array}\right)$ is the force vector relative to the transducer’s origin.

Resulting forces and torques are transmitted to the adjacent mechanical interfaces and can lead to micromotions within the taper connection. Thus, axial taper torques M_z’_ were calculated based on the torques in the center of the femoral head using a coordinate rotation around the y-axes in the taper-aligned coordinate system (x’-y’-z’) (Fig 3B).

Mathematically, the torque vector $\bar{M^{\prime }}$ is determined by calculating the cross product of the rotation matrix R_y_ and the torque vector $\bar{M}$ in the x-y-z coordinate system, as follows:

$$\bar{M^{\prime }}=\left(\begin{array}{c} M_{x^{\prime }}\\ M_{y^{\prime }}\\ M_{z^{\prime }} \end{array}\right)=R_{y}\bar{M}=\left(\begin{array}{ccc} cos\alpha & 0 & -sin\alpha \\ 0 & 1 & 0\\ sin\alpha & 0 & cos\alpha \end{array}\right)\left(\begin{array}{c} M_{x}\\ M_{y}\\ M_{z} \end{array}\right)=\left(\begin{array}{c} 0.866M_{x}-0.5M_{z}\\ M_{y}\\ 0.5M_{x}+0.866M_{z} \end{array}\right)$$ (3)

where α = 30° is the angle of rotation around the y-axes and M_z’_ is the torque around the taper axes (Fig 3B).

New ceramic-on-XPE bearings with diameters of 28, 36 and 40 mm (n = 1) were used (Table 1). All polyethylene components had been moderately crosslinked at 7.5 MRad and remelted. The acetabular components were embedded in the insert fixation with a two-component polyurethane (RenCast FC53A/B, Gößl & Pfaff, Germany) (Fig 2). Specimens had been cleaned and dried prior to assembly in the closed chamber. All air in the serum was removed before closing and sealing of the chamber.

**Table 1:** Specimens for dynamic testing.

Bearing	Head	Insert
28 mm ceramic-on XPE (n = 1)	Biolox^®^ delta (CeramTec) KK 28-12/14 M (Lot 12/589513)	Pinnacle (DePuy Synthes) ALTRX^®^ Polyethylene 28/48 (Lot 705879)
36 mm ceramic-on XPE* (n = 1)	Biolox^®^ delta (CeramTec) KK 36-12/14 M (Lot 12/2011245)	Pinnacle (DePuy Synthes) ALTRX^®^ Polyethylene 36/52 (Lot 705126)
40 mm ceramic-on XPE (n = 1)	Biolox^®^ delta (CeramTec) KK 40-12/14 M (Lot 15/52575550)	Pinnacle (DePuy Synthes) ALTRX^®^ Polyethylene 40/56 (Lot 547018)

*Note: also used for validation measurement (physical pendulum)

### 1. Demonstration of repeatability

A 36 mm ceramic-on-XPE sample bearing, identical to the one shown in Table 1, was used for the investigation of repeatability. For the single bearing, a total of five repeated measurements (1000 cycles according to the ISO 14242–1 standard for wear testing) were performed while the test chamber was dismantled and re-setup after each trial in the same way as was done for the installation of a new bearing. The maximum moment around each of the three axes x, y and z of the last cycle (999) was then calculated and compared.

### 2. Investigation of systemic effects by a hydrostatic bearing

**Fig 4:**
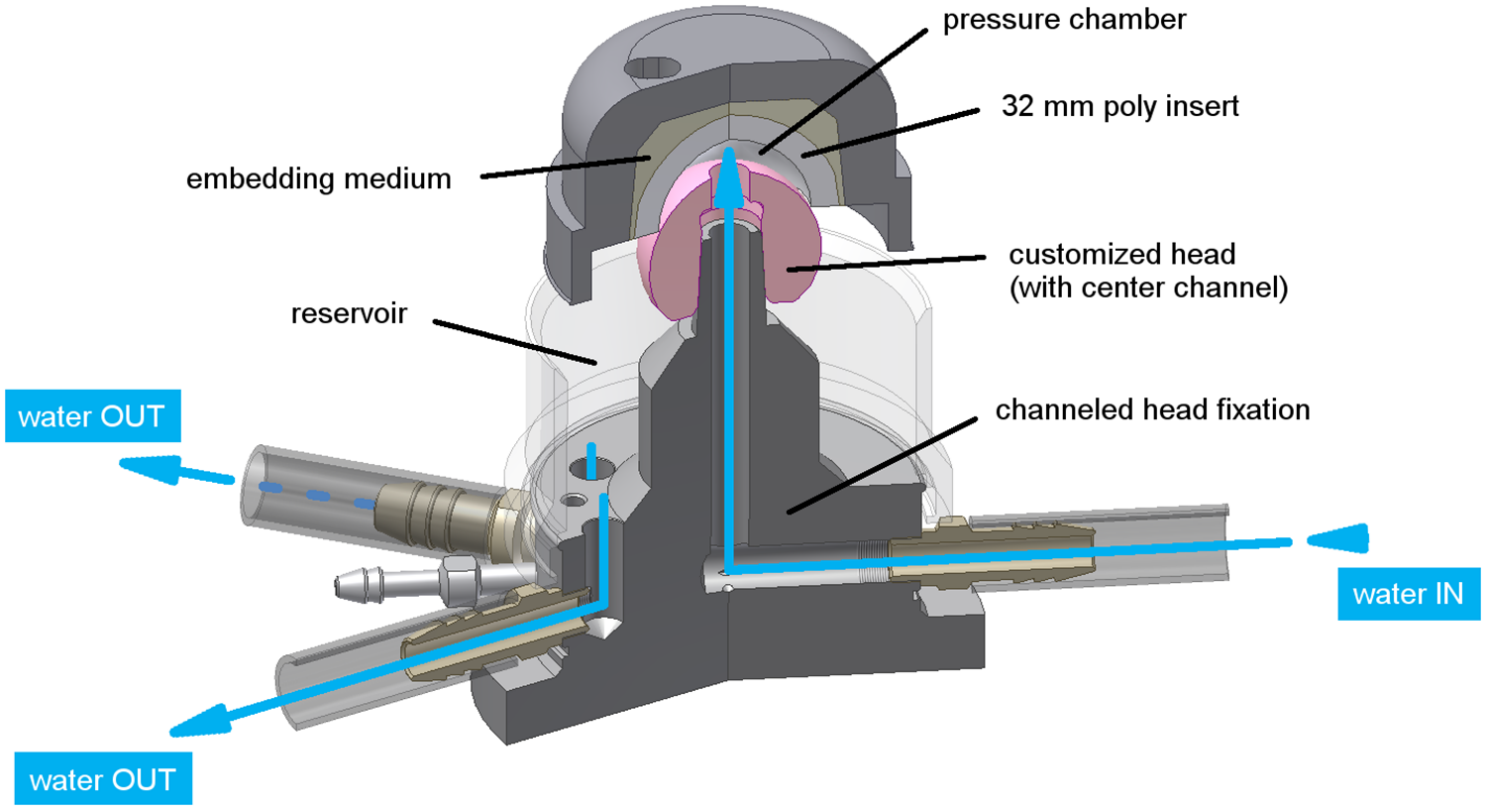
Hydrostatic bearing with water supply. Sectional view through water channels of the (ideal) bearing with fluid separated bearing surfaces.

In a highly sensitive mechanical setup, where very small forces and torques are intended to be detected, systemic errors such as mass inertia during de-/acceleration of the actuators or any system-based frictional effects must be evaluated experimentally. For the given setup, this evaluation was conducted using a hydrostatic bearing to represent the ‘perfectly lubricated’ hip bearing (Fig 4). It consisted of a 32 mm polyethylene insert and a 36 mm ceramic head with a center hole. The difference in diameter allowed for the formation of a pressurized chamber between the bearing surfaces, fed with tap water with a pressure of 5.5 bar (GMH 3150, Greisinger electronic, Germany). The water was directed through the taper body and femoral head in the gap between the articulating partners, completely separating each surface from one another.

**Fig 5:**
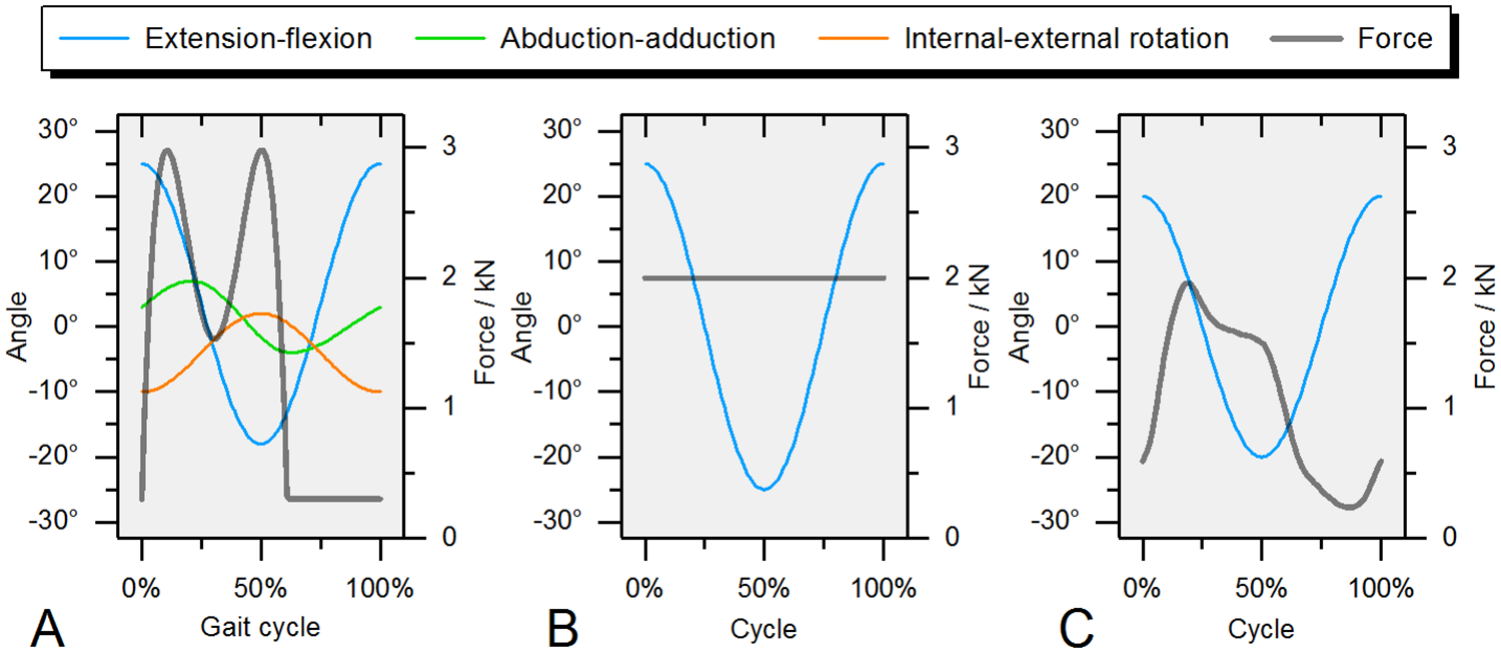
Simulator kinematics for all tested procedures. (A) ISO 14242–1, (B) extension-flexion under static load and (C) extension-flexion with a dynamic load profile.

Friction was recorded during standard ISO kinematics (Fig 5A) for ten cycles before application of the water pressure and under water supply. This resulted in complete separation of the articulating surfaces which was visible by a continuous water flow over the whole surface of the head, representing an ideal bearing. During testing, the dynamic axial force was reduced to 3 percent of the ISO load (max. load < 100 N) in order to sustain a constant fluid film between the articulating partners.

### 3. Physical pendulum with static force

**Fig 6:**
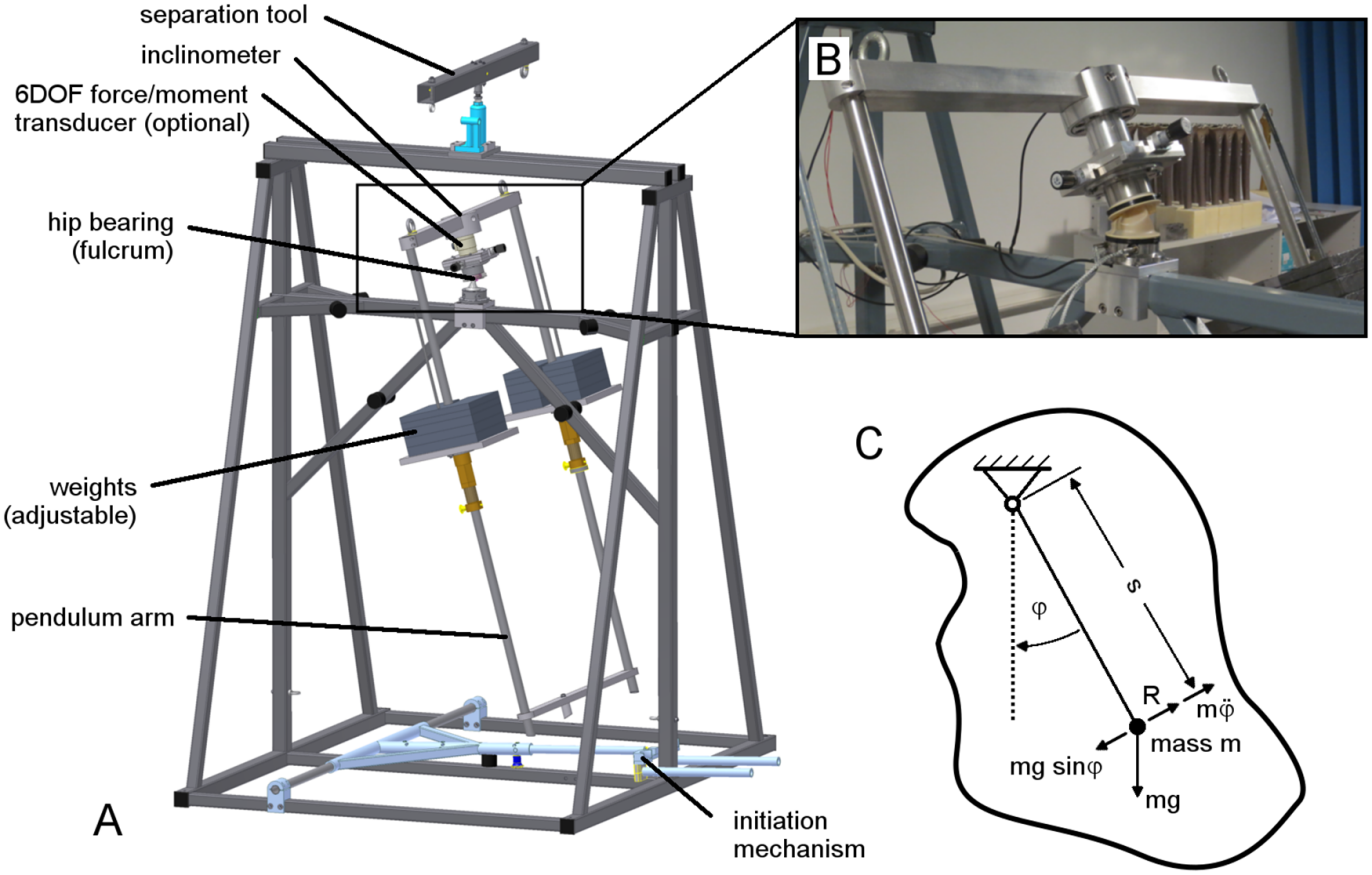
Physical pendulum. (A) Pendulum setup with 2000 N arm weight. (B) Detail view on fulcrum. (C) Theoretical model of the physical pendulum (clockwise rotation).

A friction-measuring hip simulator is a complex system which needs to be validated with a well-predictable setup that has minimal disturbance from systematic influences. Therefore, a highly reproducible physical pendulum was used, in which the hip joint was represented by the fulcrum (Fig 6). The results were compared to those from the hip simulator that had only an extension-flexion oscillation of ±25 degrees at a frequency of 0.75 Hz and a static force of 2 kN applied for a total of 60 cycles, with data from only the last 10 cycles (51–60) taken for further processing (Fig 5B and Table 2).

**Table 2:** Test matrix.

#	Procedure	Duration	Axial load	Extension (-) Flexion (+)	Abduction (-) Adduction (+)	Internal (-) Ext. (+) rot.
1	ISO 14242–1 (3D gait)	1000 cycles, 1 Hz	0.3 to 3.0 kN	-18 to +25°	-4 to +7°	-2 to +10°
2	Extension-flexion under static load	60 Cycles, 0.75 Hz	2 kN (static)	±25°	not used	not used
3	Extension-flexion with a dynamic load profile	60 Cycles, 1 Hz	0.24 to 1.95 kN	±20°	not used	not used

Hip simulator testing.

The pendulum used the same closed and temperature-controlled chamber for the specimen as mounted in the hip simulator so that any influences on the outcome were limited to the actuator system itself.

The free pendulum was not actively driven and worked by gravitation at a frequency of 0.75 Hz and a swing arm weight of 2000 N until oscillation (initial amplitude of 30 degrees) halted. The inclination angle of the pendulum arm was measured continuously at a rate of 1024 Hz (DOG2 inclinometer, ±45° dual axis, MEAS GmbH, Germany). Forces and torques were measured for the 36 mm ceramic-on-XPE bearing using the same measurement setup (6DOF transducer and X/Y table) as in the hip simulator.

In addition, the frictional torque was determined by an iterative comparison of the measured oscillation angle and the angle calculated based on the damping of the system. This theoretical approach is called a ‘best fit’ model. It has been shown that the oscillation amplitude decreases linearly over time, representing a speed-independent damping behavior caused by frictional effects in the hip bearing (fulcrum) (Fig 6). It is mechanically described by the differential equation

$$-mgs\text{ sin}\varphi \left(t\right)\pm M_{f}=\ddot{\varphi }\left(t\right)J$$ (4)

with m: total mass of the pendulum arm, g: gravitational acceleration (9.81 m/s^2^), s: distance from the head’s center to the arm’s center of gravity, φ(t): angle of rotation, M_f_: frictional torque around the pendulum axes (note that the sign of M_f_ depends on the direction of rotation), $\ddot{\varphi }$(t)J: Newton's second law of motion where $\ddot{\varphi }$(t) is the angular acceleration and J the area moment of inertia.

This can be further solved to

$$\varphi \left(t\right)=\left(\hat{\varphi }\pm A\right)\text{ cos}\left(\omega t+\varphi_{0}\right)\pm A$$ (5)

where

$$\omega =\sqrt{\frac{mgs}{J}}=2\pi f\;and\;A=\frac{M_{f}}{mgs}$$ (6)

with $\hat{\varphi }$: half cycle amplitude, φ_0_: phase shift depending of direction of rotation (0/π).

The distance s = 0.238 m was determined using the computer aided design tool (Inventor 2009, Autodesk) which calculated the location of the assembly’s center of gravity with respect to the different material properties of the pendulum components.

### 4. Extension-flexion oscillation under a dynamic force profile

An extension-flexion oscillation of ±20 degrees with a dynamic force profile based on *in vivo* data from the Orthoload database (www.orthoload.com) served as a reference for friction measurements in a driven pendulum setup from literature [33] (Table 2). In vivo forces have been calculated from the mean value of the available online data (EBL, HSR, IBL, KWL, KWR, PFL and RHR) at their mean weight (770.3 N), resulting in a peak axial force of 1.95 kN during testing (Fig 5C). Extension-flexion oscillation was performed for a total of 60 cycles, with the data of the last 10 cycles (51–60) used for further processing.

### 5. Three-dimensional friction measurement in the hip simulator

The hip simulator used for this study allowed for free programming of all kinematic channels (displacement mode) as well as the axial load. The standard kinematics and axial loading profiles, according to the ISO 14242–1, were applied for a total of 1000 cycles as a reference three-dimensional gait used for hip wear testing (Fig 5A). For the reason of the evaluation of running-in and drift effects, three friction cycles were recorded in regular intervals (after 1, 10, 25, 50, 75 and each 100 cycles) over the whole duration of testing. The last three cycles (998–1000) were used for data analysis. Maximum resultant frictional torque was calculated for each cycle and mean values of all captured cycles are taken for analysis. All results were given in percent of the test cycle where each step during ISO walking initiates with a heel strike at zero percent of the gait cycle.

## Results

**Fig 7:**
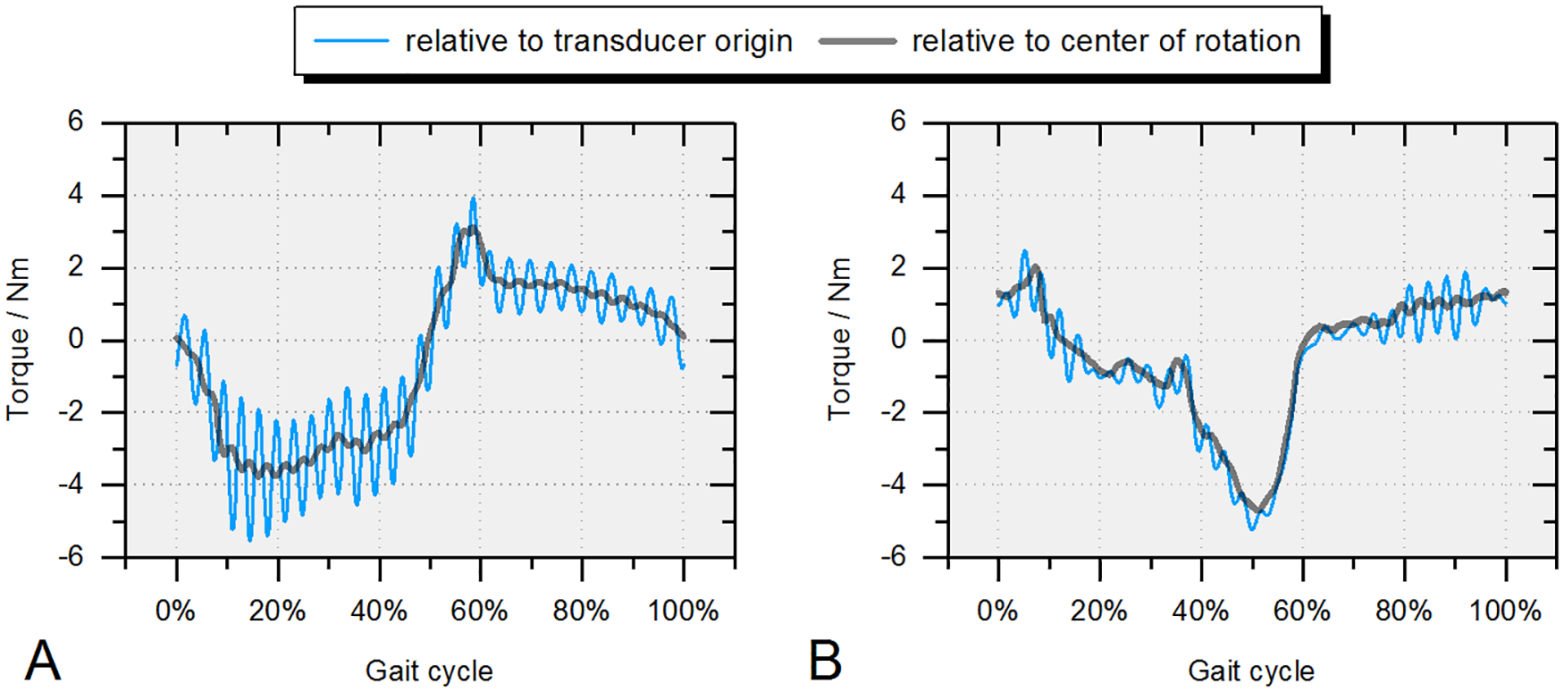
Frictional torque before (x_0_-y_0_-z_0_) and after (x-y-z) transformation of the coordinate system to the head’s center (36 mm ceramic-on-XPE). (A) M_x_ and (B) M_y_ (note that M_z_ remains unchanged as Δx = Δy = 0).

Using the modified hip simulator, torques around all axes could be measured and processed in the following way. The coordinate transformation from the *transducer origin* (center of the top plate) to the *head’s center* (Fig 3A), around which all actuator rotations are applied, resulted in a smoothening of the initial torque signals (Fig 7). This was caused by the application of the loads at a distance Δz from the transducer’s origin, mathematically evident due to the displacement matrix.

In the following section all results are given relative to the head’s center (Fig 3A) unless otherwise indicated.

### 1. Demonstration of repeatability

**Fig 8:**
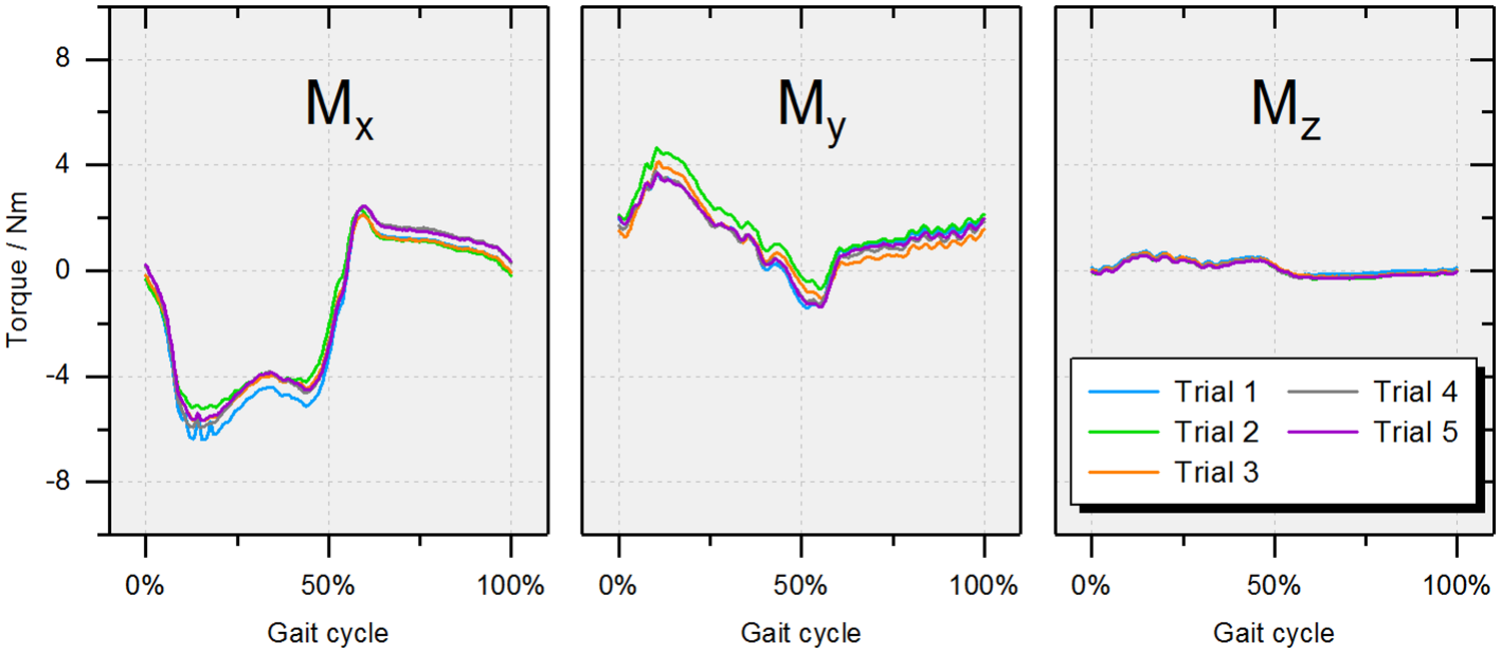
Repeated measurements for one single sample. Comparison of the results for each moment component after 1000 ISO cycles (A) M_x_, (B) M_y_ and (C) M_z_.

Results of repeated measurements for one single sample bearing are provided for each moment component in Fig 8. Regarding the maximum value over one gait cycle, M_y_ (3.95 ± 0.42 Nm) shows the largest deviation (M_x_: 2.29 ± 0.16 Nm; M_z_: 0.65 ± 0.07 Nm).

### 2. Investigation of systemic effects by a hydrostatic bearing

**Fig 9:**
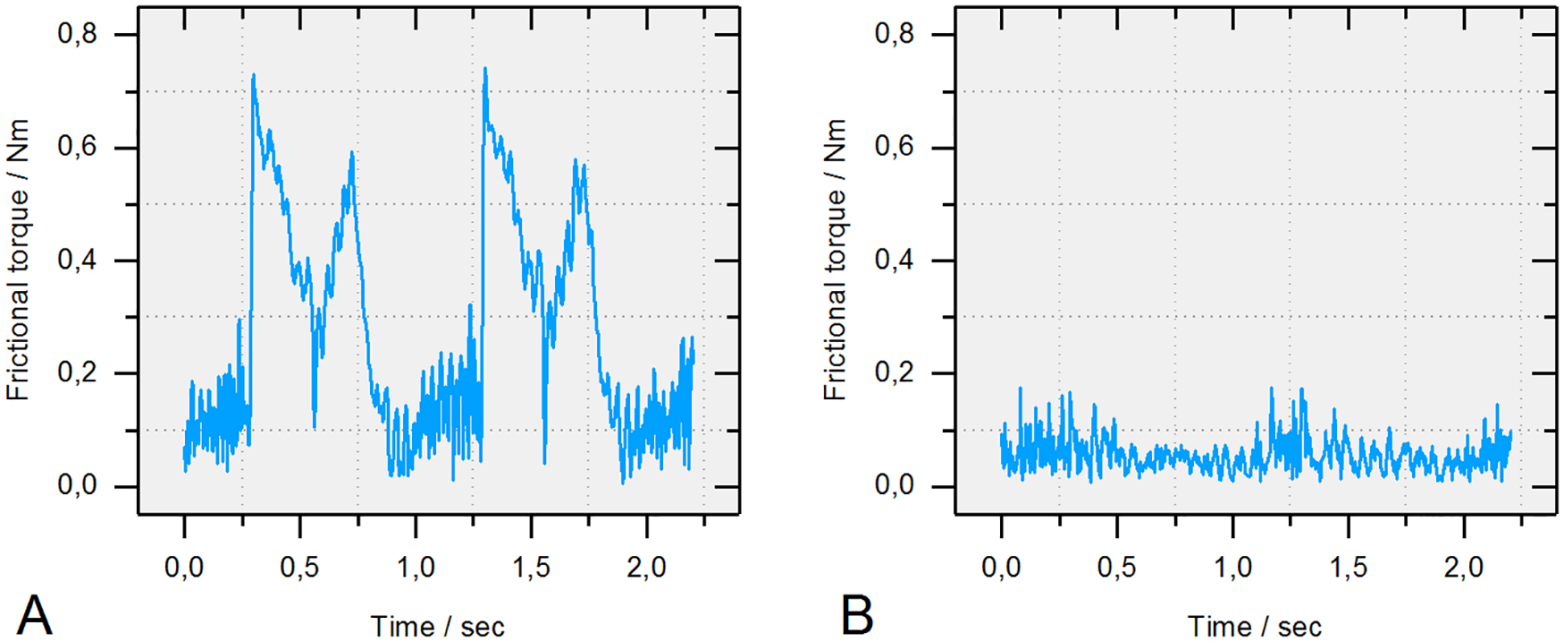
Resultant torque measured with the hydrostatic bearing (ceramic-on-polyethylene). (A) Without water pressure (dry). (B) Separation of bearing surfaces by water pressure (hydrostatic bearing).

Full separation of the articulating surfaces by the hydrostatic bearing was able to reduce friction to the signal noise and the small amount that was generated within the fluid film itself (Fig 9). As a result, the detection limit for resultant frictional torque using this setup was 0.2 Nm, as no higher systemic effects were measured during application of the water pressurized hydrostatic bearing under three-dimensional standard ISO kinematics.

### 3. Physical pendulum with static force

**Fig 10:**
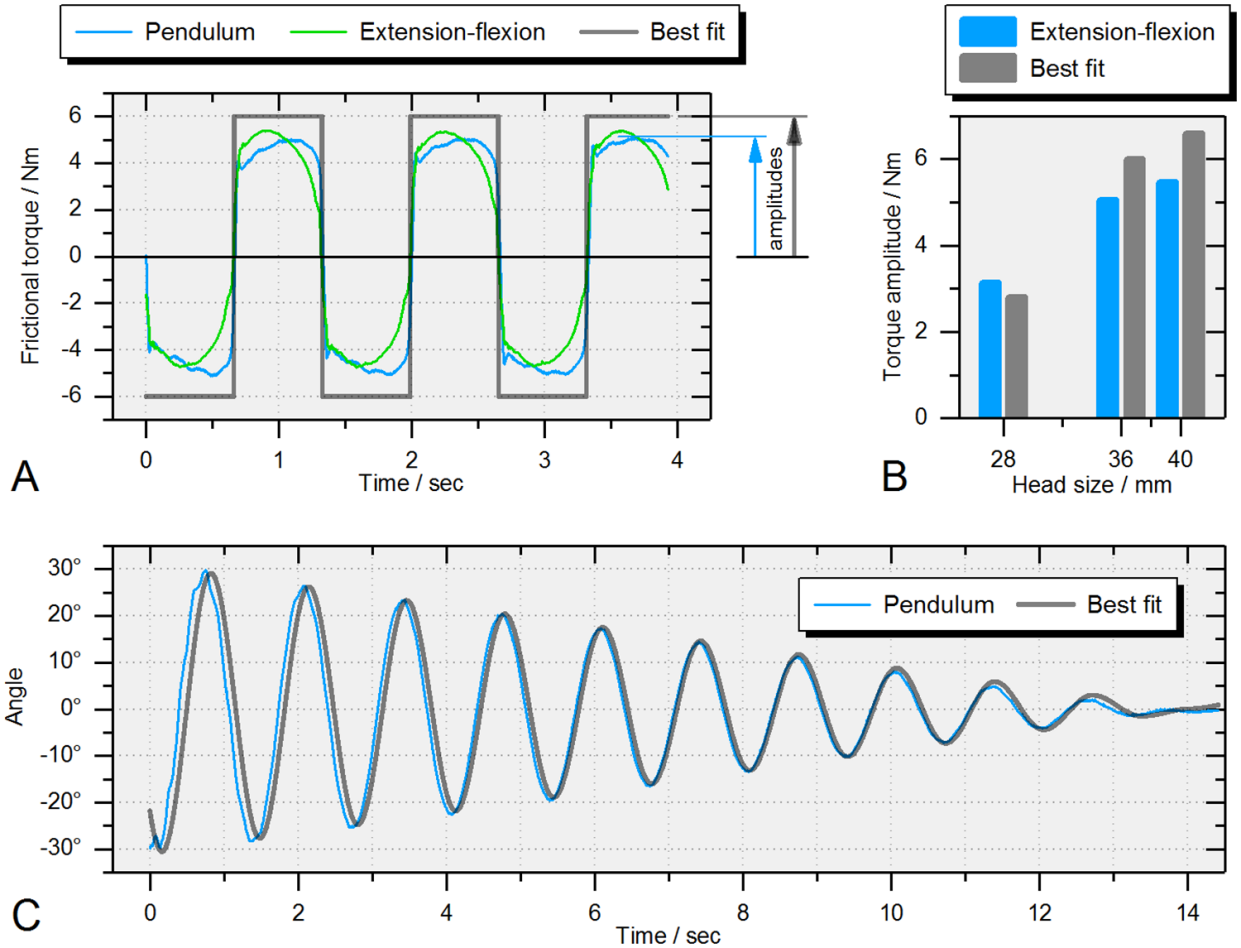
Pendulum results. (A) Comparison of friction results from the physical pendulum and the extension-flexion oscillation using the hip simulator (36 mm ceramic-on-XPE). (B) Extension-flexion (hip simulator) and best fit torque amplitudes for different head sizes. (C) Oscillation angles from experimental (Pendulum) and calculated data (Best fit, 36 mm ceramic-on-XPE).

Symmetrized torque results from the physical pendulum (36 mm ceramic-on-XPE) were in accordance with those from extension-flexion simulator testing (Fig 10A). Interestingly enough, maximal frictional torque remained constant during oscillation even though the amplitude decreased over time, which is in accordance with the theory of speed-independent damping behavior applied for the theoretical frictional torque calculation (best fit). This can also be seen by the characteristic linear decrease in angular oscillation (Fig 10C). Comparing results of torque amplitudes from the hip simulator and the best fit calculation showed slight differences but a general trend of increasing torque amplitude with larger head diameters (Fig 10B).

### 4. Extension-flexion oscillation under a dynamic force profile

**Fig 11:**
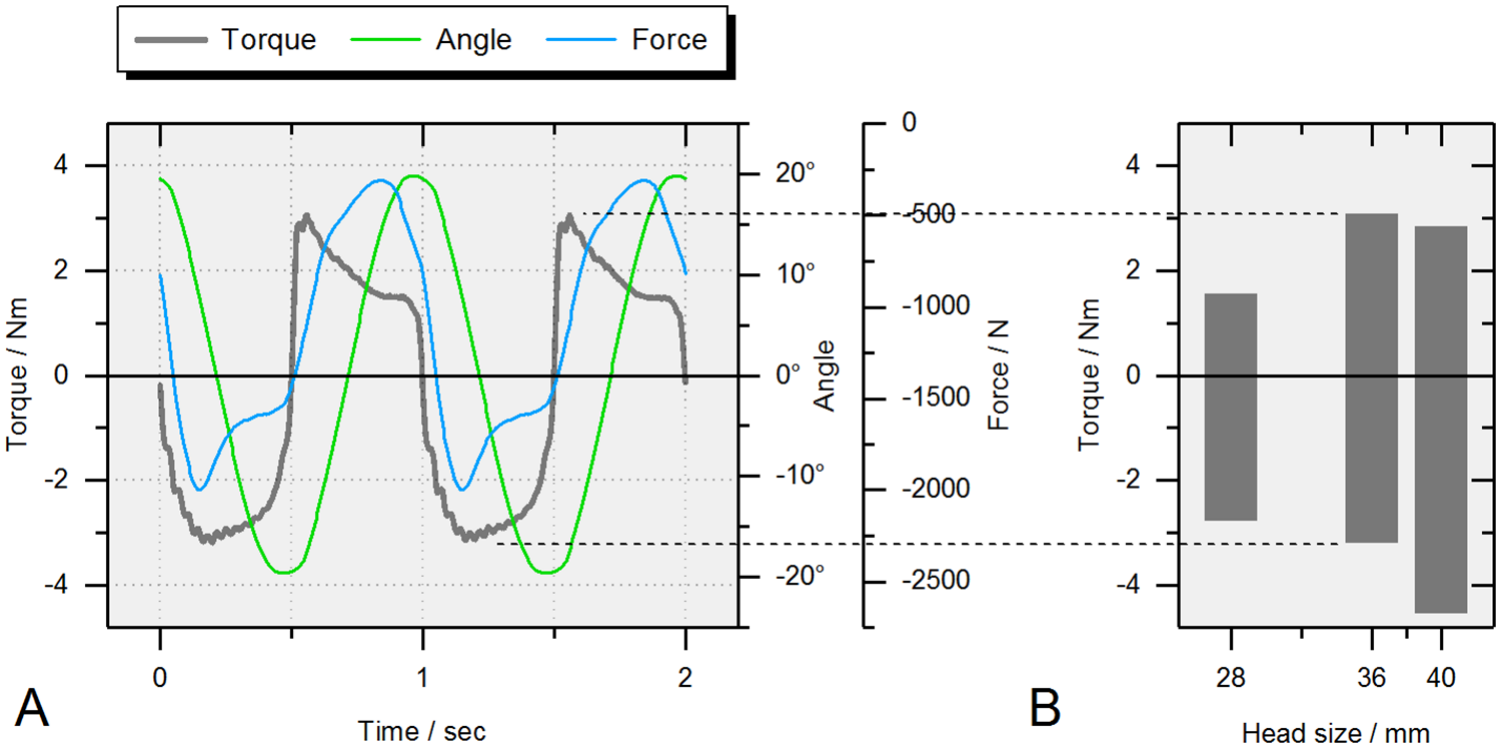
Results from extension-flexion oscillation with a dynamic force profile. (A) Frictional torque around the axis of rotation with force and motion profile (36 mm ceramic-on-XPE). (B) Min-Max torques for the investigated ceramic-on-XPE bearing sizes.

The application of a dynamic force profile and sinusoidal extension-flexion oscillation in the hip simulator resulted in an asymmetric torque pattern (Fig 11A), which is also reported in literature [33]. Peak frictional torques were measured during high axial loading and turnaround points. Analogous to the static pendulum results, frictional torque increased with larger head diameters (Fig 11B).

### 5. Three-dimensional friction measurement in the hip simulator

**Fig 12:**
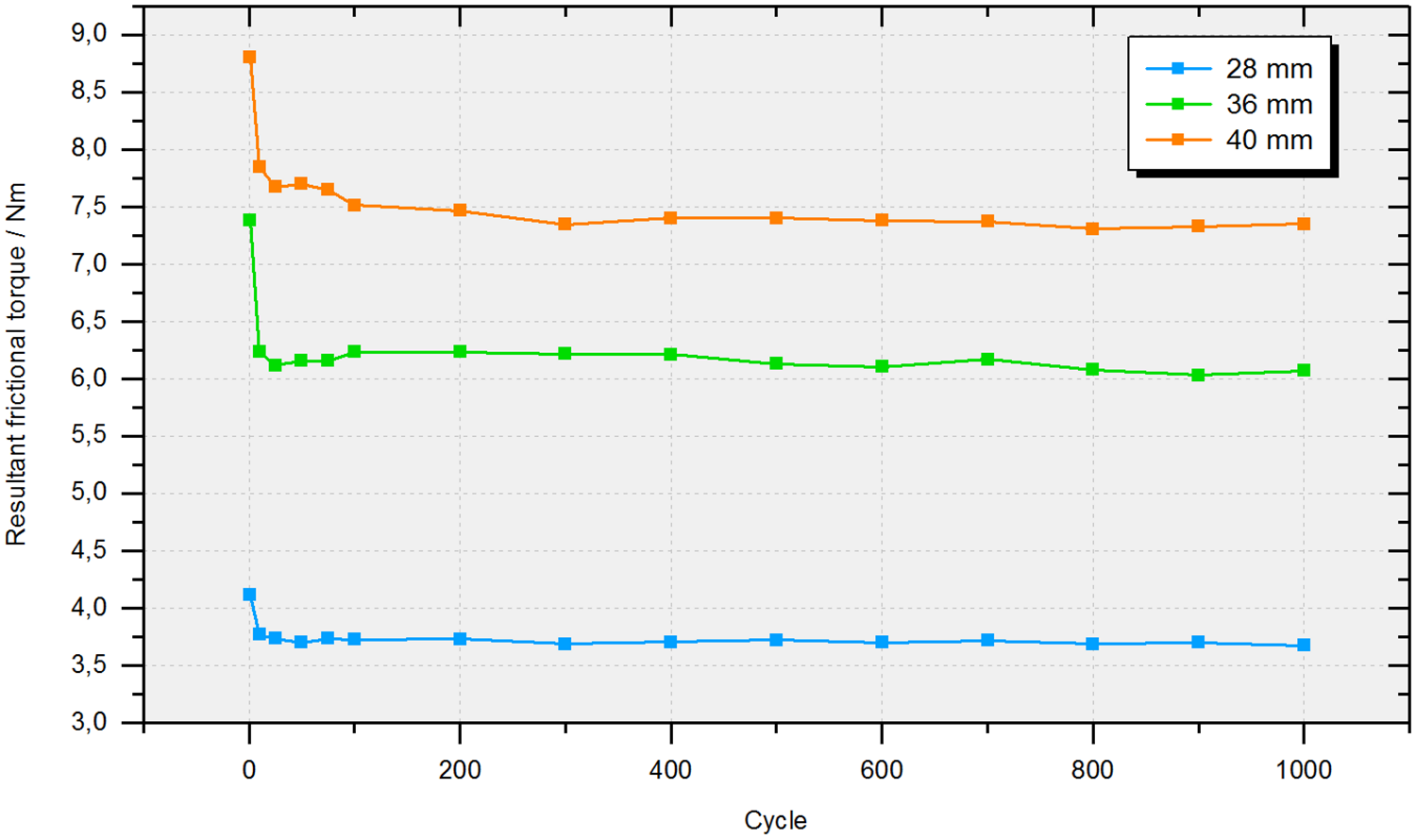
Resultant frictional torque over the total testing duration of 1000 ISO cycles. Comparison of ceramic-on-XPE bearings of 28, 36 and 40 mm nominal diameter.

In a three-dimensional analysis of friction, the resultant torque is the most important parameter, as it represents the total amount of friction which is generated over a gait cycle. The resultant is calculated by pythagorean addition of the torquesM_x_, M_y_ and M_z_ around the axes of the Cartesian coordinate system in the head’s center (Fig 3A). For the investigated ceramic-on-XPE bearings, the resultant torque was shown to reach a steady-state plateau after approximately 200 to 400 cycles (Fig 12) without any signs of drifting over the duration of testing. Thus, all data is given for the maximum of 1000 ISO cycles in the following (mean values of cycles 998 to 1000).

**Fig 13:**
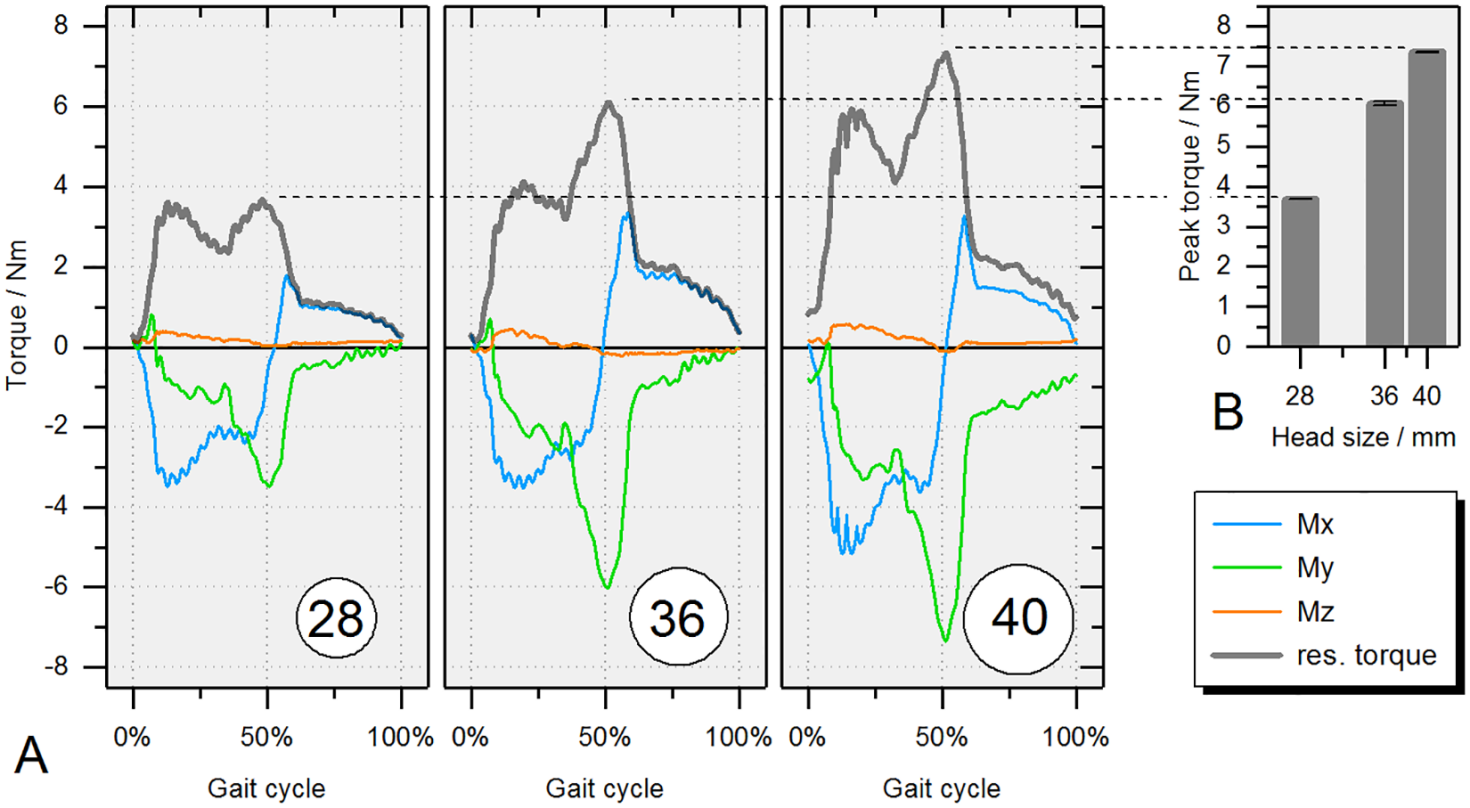
Results from the ‘normal gait’ kinematics according to ISO 14242–1 (x-y-z coordinate system in the head’s center). (A) Frictional torque for ceramic-on-XPE with bearing diameters of 28, 36 and 40 mm (cycle 999). (B) Mean maximum frictional torques and standard deviations of the last three consecutive gait cycles (997–999).

Fig 13A shows the individual as well as the resultant torque around each axis after 1000 cycles. For single rotations, the individual torques were maximal at 10 to 15 percent of the gait cycle for M_x_ and M_z_ and at 50 percent for M_x_ and M_y_ which were the instances of maximal axial force, small angular velocity in the respective channel, and maximal resultant frictional torque.

**Fig 14:**
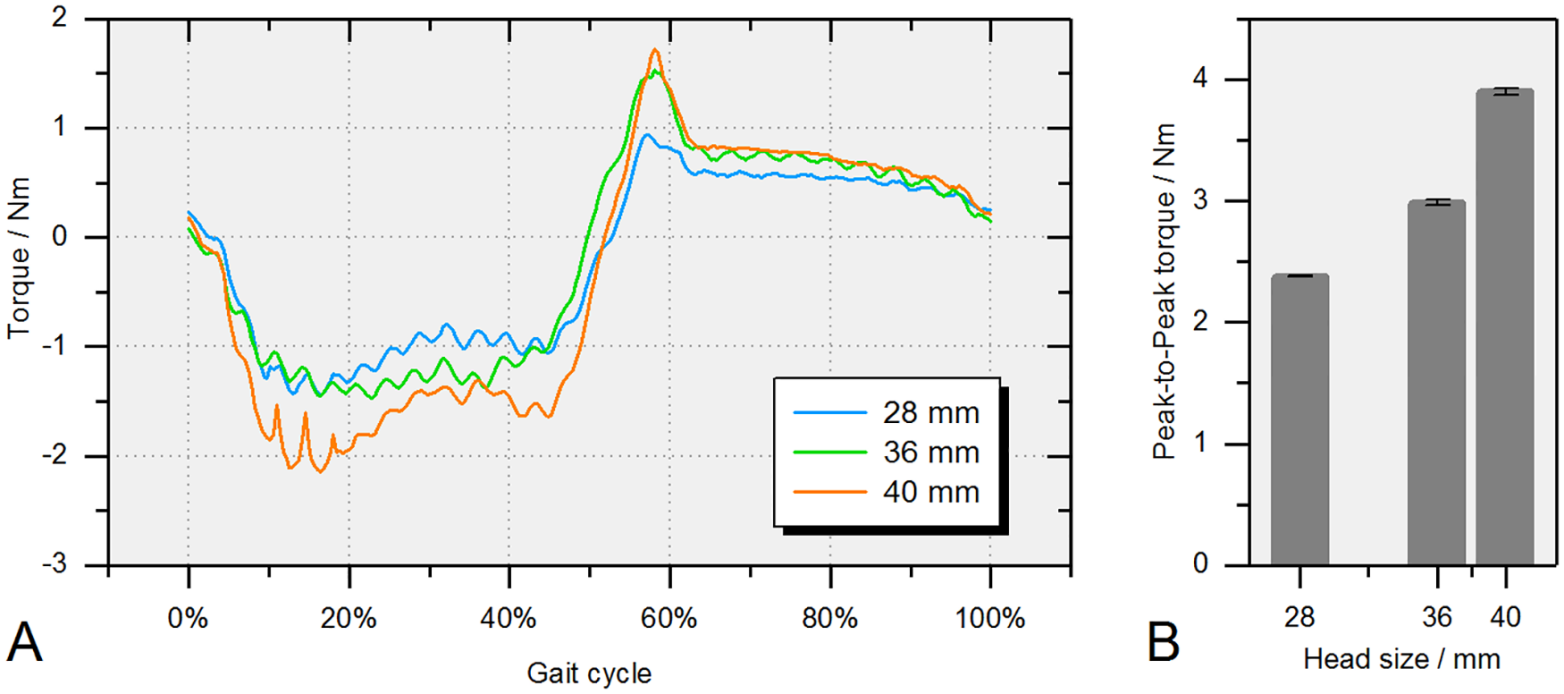
Isolated torque around the taper axes (M_z’_, Fig 3B). (A) Ceramic-on-XPE bearings of different head sizes (28, 36 and 40 mm). (B) Mean frictional taper torque peak-to-peak values (min-max) of three consecutive gait cycles.

Increasing the head size of the ceramic-on-XPE bearing resulted in an increase of the resultant frictional torque (Fig 13B). The same trend of increased friction with larger bearing diameters was also seen for the torque around the taper axis (Fig 3B), ranging from -1.44 to 0.94 Nm for the 28 mm bearing, -1.47 to 1.54 Nm for the 36 mm bearing and -2.14 to 1.72 Nm for the 40 mm bearing (Fig 14A). This resulted in an increase in torque peak-to-peak values (Fig 14B).

## Discussion

Measurements after repeated dismantling and setup generated reproducible results for all moments’ components with M_y_ having the largest deviation between the repeated trials. This may be due to the deflection of the head fixation during dynamic loading. Verification with the hydrostatic bearing (systemic effects < 0.2 Nm) and validation by the physical pendulum has shown that three-dimensional friction measurements using a modified hip simulator are able to provide data that give a comprehensive overview of how a hip bearing functions under complex kinematics and dynamic forces [34]. The small differences between the theoretical best fit, based on the damping behavior of the physical pendulum, and the hip simulator measurements may be related to differences in the approaches, e.g. vibrations or minimal axial rotation during oscillation of the pendulum, differences in the computer-calculated and true distance between the fulcrum and the center of gravity of the pendulum arm, and the centrifugal force during pendulum oscillation which is not taken into account during hip simulation (static uniaxial force application).

Nevertheless, the best fit results are well comparable to those found during experimental testing in the pendulum and the hip simulator, where the latter two are very consistent. Thus, the more theoretical pendulum approach without the need for a force and torque measurement device may be interesting for fast screening friction tests that do not require a three-dimensional approach and complex kinematics or dynamic forces.

According to the literature on friction measurements summarized in Table 3, absolute results on frictional torque under a static axial load are not common. In an early study, Ma et al. [35] used a driven pendulum at a load of 890 N for a metal-on-polyethylene bearing. Assuming a linear increase of the frictional torque with larger head diameters and higher axial loads, this resulted in an average frictional torque of 2.92 Nm and 5.53 Nm at 28 mm and 40 mm head diameter at a load of 2000 N, which is in accordance with the present results for the ceramic-on-XPE bearing. Kaddick et al. [31] reported a mean frictional torque for a new ceramic-on-polyethylene bearing of 4.5 Nm and a slight decrease for a used one (after 5 million wear cycles), which is still higher than in this study, although the very early results from O’Kelly et al. [21] on a small 22.25 mm metal-on-polyethylene bearing are consistent with the present results.

**Table 3:** Literature data on friction measurements on total hip replacements with polyethylene bearings.

Authors	Year	Ref	Bearing	Head size	Kinematics	Load	Res. torque^1^
*Single rotation under static load*							
O’Kelly et al.	1977	[21]	MoP	22.25 mm	free pendulum^2^	2000 N	3 Nm^3^^,^^4^
Ma et al.	1983	[35]	MoP	28 mm 43 mm 51 mm	driven oscillation (±30°)	890 N	1.3 Nm^3^ 2.75 Nm^3^ 3.2 Nm^3^
Kaddick et al.	2015	[31]	CoP (new) CoP (used)	28 mm	extension-flexion hip simulator (±24°)	2000 N	4.5 Nm 4.0 Nm^3^
present study	2017		CoP	28 mm 36 mm 40 mm	extension-flexion hip simulator (±25°)	2000 N	3.14 Nm^5^ 5.06 Nm^5^ 5.47 Nm^5^
*Single rotation with a dynamic load profile*							
Brockett et al.	2007	[22]	MoP CoP	28 mm	driven oscillation (±25°)	60%-sine max = 2000 N	2.3 Nm 2.2 Nm
Bishop et al.	2007	[36]	MoP	28 mm	driven oscillation (±20°)	in vivo max = 2000 N^2^	3.69 Nm^3^^,^^4^
Bishop et al.	2008	[33]	MoP	28 mm	driven oscillation (±20°)	in vivo max = 2000 N	4.1 Nm
Bishop et al.	2012	[37]	CoP	36 mm	driven oscillation (±20°)	in vivo max = 2000 N	3.06 Nm^3^^,^^4^
present study	2017		CoP	28 mm 36 mm 40 mm	extension-flexion hip simulator (±20°)	in vivo max = 1960 N	2.8 Nm 3.2 Nm 4.5 Nm
*Three-dimensional hip articulation (no orbital rocking simulators)*							
Damm et al.	2013	[38]	CoP (3 mo.)	32 mm	in vivo ‘Normal walking’	in vivo data max = 2036 N^6^	1.81 Nm^6^
Damm et al.	2015	[39]	CoP (3 mo.) CoP (12 mo.)	32 mm	in vivo ‘Normal walking’	max = 2274 N^6^ max = 2185 N^6^	2.11 Nm^6^ 1.55 Nm^6^
Kaddick et al.	2015	[31]	CoP (new) CoP (used)	28 mm	ISO 14242–1	ISO 14242–1 max = 3000 N	-1.7 to 3.7 Nm^7^ -1.5 to 1.2 Nm^7^
Haider et al.	2016	[26]	MoP	40 mm	ISO 14242–1	ISO 14242–1 max = 3000 N	3.6 Nm^3^^,^^4^
present study	2017		CoP	28 mm 36 mm 40 mm	ISO 14242–1	ISO 14242–1 max = 3000 N	3.77 Nm 4.77 Nm 5.36 Nm
present study	2017		CoP	28 mm 36 mm 40 mm	ISO 14242–1	ISO 14242–1 max = 3000 N	-1.5 to 0.9 Nm^7^ -1.5 to 1.5 Nm^7^ -2.2 to 1.7 Nm^7^

MoP: metal-on-polyethylene; CoP: ceramic-on-polyethylene

^1^ Maximum value over a gait cycle (unless otherwise specified);

^2^ Not further specified;

^3^ Data taken out from graph;

^4^ Calculated based on friction factor f and axial load L;

^5^ Torque amplitude during oscillation;

^6^ Calculated for the average patient (bodyweight = 821 N);

^7^ Data given around taper axis.

It is supposed that the ‘squeeze-film’ mechanism during dynamic joint loading may be important to build up a load bearing lubrication film as seen in the human synovial joint [34]. Thus, pendulum testing with a dynamic loading, corresponding to the extension-flexion oscillation under a dynamic force profile in this study, has been performed by several authors even though their setups and methods differ in kinematics and the measurement technique of the frictional torque as well as the individual force profile. Bishop et al. [37] reported a maximum frictional torque of 3.06 Nm for a 36 mm ceramic-on-polyethylene bearing, which is in accordance with the maximum frictional torque in this study. Compared to measurements of a metal-on-polyethylene bearing of an identical size by the same authors, slightly increased frictional torque (3.69–4.1 Nm) was observed [33, 36], exhibiting the influence of the head material articulating against polyethylene. Brocket et al. [22] reported a smaller torque which may be due to the difference in the applied kinematics and the force profile. This effect can also be seen when comparing the simulator results from the present study for the extension-flexion oscillation, where the maximum frictional torque was smaller for a dynamic force profile compared to the oscillation at a constant peak force.

The results from the three-dimensional friction measurements were very consistent with the predicted lubrication regime in numerical models [13], where the lambda ratio had been calculated as a parameter for the lubrication film height relative to the topographical characteristics of the articulating surfaces. It has been shown that this parameter is minimal (representing poor lubrication) at about 50 percent of the gait cycle which corresponds well to the instance of maximal frictional torque measured in this study. Analogous to wear assessment of results from different simulator concepts, the comparison of absolute friction values with measurements from other three-dimensional approaches must address the differences in the methods used for friction assessment. To that point, friction measurements by an orbital rocking simulator are normally taken around the single vertical internal/external rotation axes and are thus not easy to compare with resultant torques from three-dimensional measurements [27–30, 40, 41]. Kaddick et al. performed three-dimensional friction testing on new 28 mm ceramic-on-polyethylene bearings and those that had already run a 5 million wear test [31]. Considerably increased torques around the taper axes were reported for the new bearing after 240 ISO cycles. Taper torques from the used components compare well with the results around the taper axis in this study (-1.5 to 0.9 Nm). Interestingly, the reduction in frictional torque of the worn-in bearing was much higher for three-dimensional ISO simulation than for an extension-flexion oscillation under a static load [31]. From the large difference in running-in and steady-state torque, it appears that the condition of the polyethylene bearing used for friction assessment plays a significant role and may also account for the small differences between these studies. However, in both cases, torque values around the taper axes during continuous ISO testing are small compared to those that are necessary to initiate mechanical loosening [42, 43]. Haider et al. [26] performed friction measurement on 40 mm metal-on-polyethylene bearings in a standard hip simulator intentionally used for wear testing. They reported a lower maximum frictional torque compared to the 40 mm ceramic-on-XPE bearing in the present study. Aside from the different head materials used, the difference in torque values may be related to the different simulator concepts and measurement strategies. Using instrumented hip implants with a ceramic-on-polyethylene bearing and a nominal diameter of 32 mm, Damm et al. [39] reported a decrease in peak friction torques from 3 to 12 months in vivo. On the other hand, earlier results from a slightly different cohort showed a large variation in torque measurement after 3 months post-operatively which is also shown by inter-individual differences up to 450 percent [38, 39].

## Conclusions

Measuring friction in a hip simulator is a challenging task that requires special attention to any confounding effects that may appear during dynamic three-dimensional testing. This study has shown that a standard simulator can be adapted to reduce alignment, crosstalk and mass inertia effects. This was achieved using an aerostatic bearing for lateral force compensation and a high-precision multi-axes transducer. The setup was validated with a classical pendulum and verified by a ‘perfectly lubricated’ hydrostatic hip bearing. Results from the three-dimensional friction measurements make it possible to see how a hip bearing works during complex kinematics and loading in an experimental setup. This may help to investigate frictional effects for different bearing materials and designs even under compromised conditions, e.g. higher cup inclinations or during gait initiation after a resting period.

Therefore, it is concluded that the investigation of friction measurements is able to expand pre-clinical testing over the established wear testing according to the standards. Doing so, an early identification of high frictional torques such as those that were reported for failed metal-on-metal bearings, may help to prevent future failure and the use of potentially deficient hip bearings.
